# Gastrodin Inhibits Bacterial Biofilm Formation, Thereby Activating the Antibacterial Activity of Antibiotics

**DOI:** 10.3390/molecules31122123

**Published:** 2026-06-16

**Authors:** Ji-Hyun Yoon, Yeo-Jin Kim, Ki-Young Kim

**Affiliations:** 1 Graduate School of Biotechnology, Kyung Hee University, Giheung, Yongin 17104, Gyeonggi-do, Republic of Korea; hayop0909@khu.ac.kr; 2 Graduate School of Business Administration, Sungkyul University, 53 Sungkyuldaehak-ro, Anyang-si 14097, Gyeonggi-do, Republic of Korea

**Keywords:** gastrodin, pathogenic bacteria, biofilm formation, synergistic antibacterial effects

## Abstract

(1) Background: The increasing antibiotic resistance of pathogens is necessitating new therapies that target virulence factors. Virulence factors include biofilm formation, which is a key pathogenic factor involved in bacterial pathogenicity and resistance. (2) Methods: Initially, biofilm formation assays were performed to screen the biofilm formation inhibition effects of gastrodin. A bacterial growth assay was performed to examine the synergistic effects and qRT-PCR was performed to identify the underlying molecular regulatory mechanisms. (3) Results: Gastrodin inhibits biofilm formation by bacteria such as *E. faecalis* (IC_50_ = 1.56 μg/mL), *E. faecium* (IC_50_ = 0.19 μg/mL), *S. aureus* (IC_50_ = 6.25 μg/mL), *C. acnes* (IC_50_ = 0.78 μg/mL), *S. sobrinus* (IC_50_ = 12.5 μg/mL), *P. aeruginosa* (IC_50_ = 25.00 μg/mL), and *E. coli* (IC_50_ = 25. 10 μg/mL) without directly affecting bacterial growth, as shown by bacterial growth assay. Gastrodin also reduced the expression of cytolysin genes (*cylLS*, *cylR2*, and *cylM*), quorum sensing genes (*fsrB*, *fsrC*, *gelE*, *ebpA*, *ebpB*, *acm*, *scm*, and *bps*) and biofilm virulence genes (*esp*) as shown by qRT-PCR analysis and exhibited dramatic synergistic antibacterial effects in the growth assay. (4) Conclusions: These results suggest that gastrodin may be a promising novel antibacterial adjuvant for biofilm-related bacterial infections, but further experiments, including in vivo assays, are still needed.

## 1. Introduction

Antibiotic resistance is the capability of bacteria to survive and grow despite exposure to antibiotics that would normally inhibit their growth or kill them, and it has emerged as a serious global health threat due to the rapid evolution and spread of resistant strains. The misuse or overuse of antibiotics in human medicine, veterinary practice, and agriculture accelerates this process by creating strong selection pressure and frequent exposure to subtherapeutic doses, thereby favoring survival of resistant bacteria. As a result, the global escalation of antibiotic resistance has now surpassed the pace at which new antimicrobial agents are being discovered and developed. Infections become harder and costlier to treat, leading to prolonged illness, higher risk of complications, and increased mortality, especially in vulnerable patients or in healthcare settings [1,2,3].

Because the pipeline for new antibiotics has not kept pace with the rapid spread of resistance, alternative therapeutic strategies are urgently needed. One promising approach is the development of anti-virulence agents that attenuate bacterial pathogenicity without necessarily exerting direct bactericidal pressure. Among virulence-associated traits, biofilm formation is especially important because biofilms promote bacterial persistence, protect pathogens from host immune responses, and reduce susceptibility to antibiotics. Therefore, inhibition of biofilm formation has emerged as an attractive strategy to restore or enhance the efficacy of existing antimicrobial agents [4,5,6].

Biofilms are highly structured microbiological communities enclosed within an extracellular polymeric matrix, widely distributed across environmental and clinical settings [7]. They can form on the epithelium, organs such as the lungs and heart, and implanted medical devices such as central venous and urinary catheters, intrauterine devices and prosthetic heart valves and can confer many advantages to bacteria, including resistance to antibiotics and immune cells [8,9]. For example, enterococci, which inhabit the gastrointestinal tract of metazoans, rarely cause infections in healthy individuals [10]. However, they can cause infections in intensive care unit patients, those with compromised immune systems, and those receiving immunosuppressive medications for conditions such as malignancy and neutropenia [11]. The ability of these enterococci to form biofilms influences their clearance by immune cells, making them a key factor in causing infections such as endocarditis, as well as infections of the nervous system, urinary tract, and bloodstream [8,9,12].

Microbial resistance to antibiotics in biofilms occurs for the following reasons. First, antibiotics cannot penetrate the biofilm, preventing them from affecting the bacteria within [13]. This is because the positively charged antibiotics bind to the negatively charged biofilm matrix due to the extracellular matrix’s electrical charge, making it difficult for antibiotics to penetrate the biofilm [14]. Second, the structure of the biofilm creates concentration gradients of oxygen and nutrients [15]. This microenvironment induces a subpopulation of bacteria to enter a dormant, metabolically inactive state. These states exhibit high tolerance to conventional antibiotics that target active cellular processes, thereby reducing overall susceptibility. This can be a significant factor in reducing susceptibility to antibiotics, especially those whose antimicrobial mechanisms are closely linked to microbial growth. For example, oxygen deprivation within a biofilm can lead to decreased energy metabolism, which reduces the influx of antibiotics and, thus, lowers antibiotic susceptibility [8,16,17].

Biofilm development is regulated by complex signaling networks, among which quorum sensing plays a central role [18]. Quorum sensing enables bacteria to sense population density through secreted signaling molecules and to coordinately regulate gene expression when a threshold concentration is reached [19]. This regulatory system controls the expression of genes involved in adhesion, extracellular matrix production, and virulence factor synthesis, ultimately promoting biofilm maturation and maintenance [20]. Because quorum sensing governs key steps in biofilm development, it represents an important therapeutic target for anti-biofilm intervention [16,17,21].

**Figure 1:**
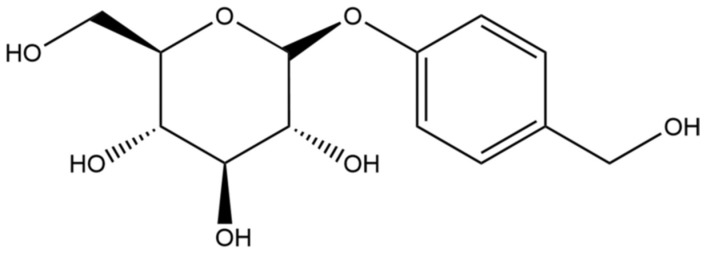
Chemical structure and molecular formula (C_13_H_18_O_7_) of gastrodin.

Gastrodin (Figure 1) is a natural compound extracted from *Gastrodia elata* and is known as a β-D-glucoside of 4-hydroxybenzyl alcohol [22,23]. It has been widely studied for its neuroprotective, cognitive-enhancing, antidepressant, anti-inflammatory, and antioxidative properties [23,24]. Recent studies have reported the antimicrobial potential of *Gastrodia elata* extracts and gastrodin derivatives [25,26]. However, its potential antibacterial and anti-biofilm activities have not been sufficiently investigated. Given its reported biological activities and relatively low toxicity, gastrodin may represent a promising candidate for repurposing as an anti-virulence agent.

In this study, we investigated whether gastrodin could inhibit biofilm formation in several clinically important bacterial species, including *Enterococcus faecalis*, *Enterococcus faecium*, *Staphylococcus aureus*, *Cutibacterium acnes*, *Streptococcus sobrinus*, *Pseudomonas aeruginosa*, and *Escherichia coli*. We also examined whether gastrodin could enhance the antibacterial activity of conventional antibiotics and explored the possible molecular basis of its anti-biofilm effect through qRT-PCR analysis of quorum sensing- and biofilm-related genes. In addition, we assessed its cytotoxicity in human-derived cells to evaluate its potential as a therapeutic adjuvant.

## 2. Results

### 2.1. Gastrodin Inhibited Bacterial Biofilm Formation

**Figure 2:**
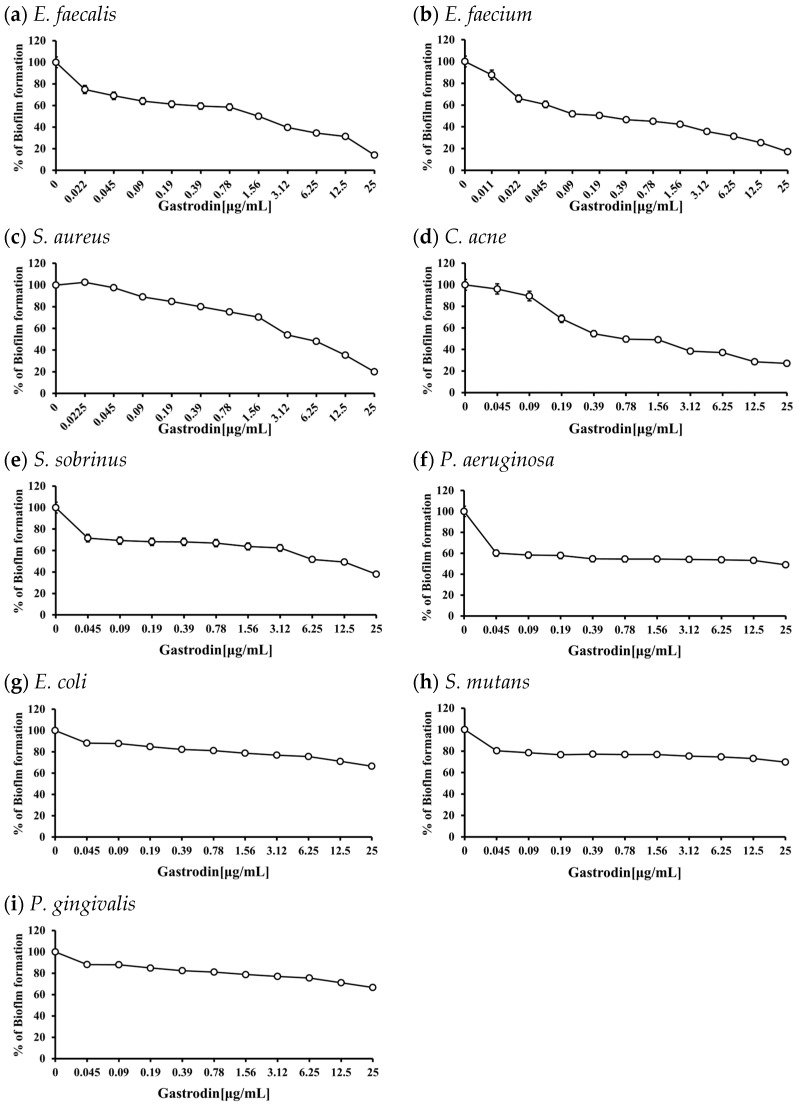
Gastrodin inhibited biofilm formation in *E. faecalis*, *E. faecium*, *S. aureus*, *C. acnes*, *S. sobrinus*, *P. aeruginosa*, and *E. coli*. Biofilm formation was induced in appropriate medium indicated in the Methods section for 24 h.

Gastrodin inhibited the bacterial biofilm formation activity against *E. faecalis*, *E. faecium*, *S. aureus*, *C. acnes*, *E. coli*, *S. sobrinus*, *P. aeruginosa*, *P. gingivalis*, and *S. mutans* (Figure 2). Gastrodin had the strongest effect on *E. faecium* (IC_50_ = 0.19 μg/mL), inhibiting more than half of biofilm formation. It also effectively inhibited biofilm formation in *C. acnes* (IC_50_ = 0.78 μg/mL), *E. faecalis* (IC_50_ = 1.56 μg/mL), *S. aureus* (IC_50_ = 6.25 μg/mL), *S. sobrinus* (IC_50_ = 12.5 μg/mL), *P. aeruginosa* (IC_50_ = 25.00 μg/mL), and *E. coli* (IC_50_ = 25.10 μg/mL). *C. acnes* was also susceptible to gastrodin since 0.78 μg/mL of gastrodin was sufficient to inhibit more than half of biofilm formation. Biofilm formation of *E. faecalis*, *S. aureus*, *S. sobrinus*, and *E. coli* was also inhibited by gastrodin. Gastrodin only showed a weak but observable effect on *P. aeruginosa*, *P. gingivalis*, and *S. mutans*.

### 2.2. Gastrodin Synergistically Activated Antibacterial Activity with Commercialized Antibiotics

Among the tested bacteria, *E. faecalis* and *E. faecium* were selected for subsequent synergistic and gene expression studies because they exhibited the strongest biofilm inhibition (lowest IC_50_ values) and possess the well-characterized *fsr* quorum sensing system, which is the primary mechanistic target of this investigation. Furthermore, *Enterococcus* spp. are among the most clinically significant nosocomial pathogens associated with biofilm-related infections. To determine whether gastrodin, which inhibits biofilm formation, can alter bacterial sensitivity to existing antibiotics, its antibacterial activity against *E. faecium* and *E. faecalis* was confirmed by mixing it with existing antibiotics.

Gastrodin with 1.56 µg/mL ampicillin (Amp), 0.78 µg/mL oxytetracycline (Oxy), 100 µg/mL vancomycin (Van), or 0.39 µg/mL streptomycin (Stre) was added to 24 h biofilm cultures at the indicated concentrations.

Treatment with gastrodin alone did not significantly inhibit bacterial growth compared to the control group. However, combination treatment with gastrodin and ampicillin decreased the survival rates of *E. faecalis* and *E. faecium* to 3.52% and 62.3%, compared to 38.76% and 86.7% for ampicillin treatment alone, respectively. Also, compared to the groups treated with oxytetracycline, vancomycin, and streptomycin alone, combination treatments with gastrodin also reduced the survival rates of *E. faecalis* to 49.04%, 42.3%, and 43.24%, respectively, and the survival rates of *E. faecium* to 43.05%, 42.88%, and 38.92%, respectively.

**Figure 3:**
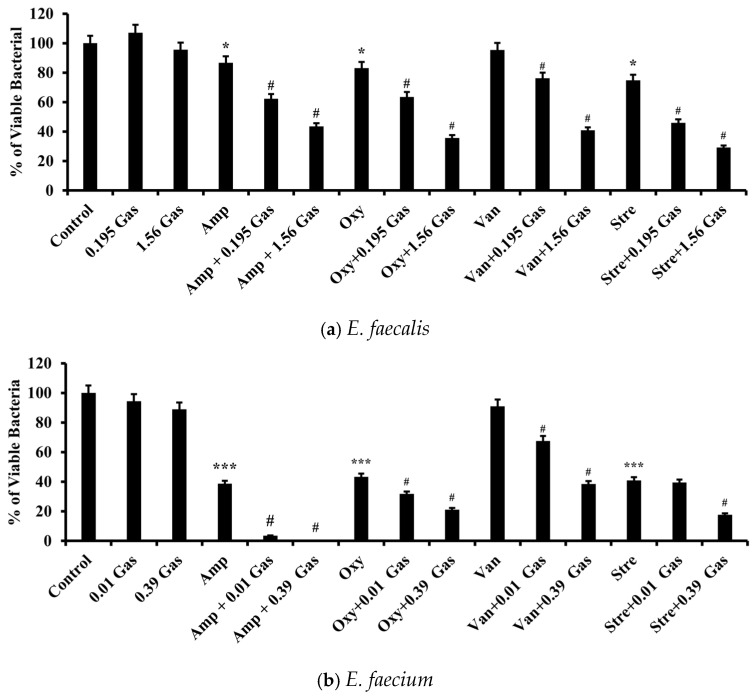
Gastrodin synergistically induced antibacterial activity with commercial antibiotics against *E. faecalis* and *E. faecium.* (**a**) The synergistic antibacterial effect between gastrodin and antibiotics against *E. faecalis*. (**b**) The synergistic antibacterial effect between gastrodin and antibiotics against *E. faecium*. [Ampicillin (Amp), Oxytetracycline (Oxy), Vancomycin (Van), and Streptomycin (Stre); * *p* < 0.05, *** *p* < 0.001, ^#^ *p* < 0.05 to the control].

These results suggested that the antibacterial effect is greater when used in combination with gastrodin than when used alone, which means that biofilm formation is inhibited by gastrodin, improving the antibacterial efficacy of the antibiotics (Figure 3).

### 2.3. Gastrodin Did Not Influence the Viability of the Mammalian Originated Cell Line

To confirm the mammalian-derived cell toxicity caused by gastrodin, the MTT assay was performed. In this process, 10^4^ T24 cells and RAW 264.7 cells were added per well in RPMI 1640 medium and DMEM medium, respectively, containing the indicated concentration of gastrodin (0–100 μg/mL) and cultured for 24 h.

**Figure 4:**
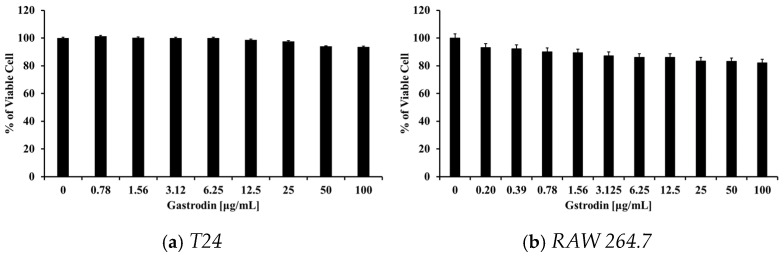
Gastrodin did not influence the viability of human-derived T24 cells and macrophages. (**a**) Gastrodin did not exhibit cellular cytotoxicity against T24 cells (**b**). Gastrodin did not exhibit cellular cytotoxicity against RAW 246.7.

Treatment of T24 cells and RAW 264.7 cells with gastrodin did not show any effect on cell growth (Figure 4). Therefore, these results suggest that gastrodin has no harmful effect on mammalian cells.

### 2.4. Gastrodin Suppressed the Expression of Biofilm Formation and Quorum Sensing-Related Genes in E. faecalis and E. faecium

To understand the molecular basis of biofilm formation inhibition, the expression of genes related to biofilm-associated factors and the QS system was tested via qRT-PCR.

**Figure 5:**
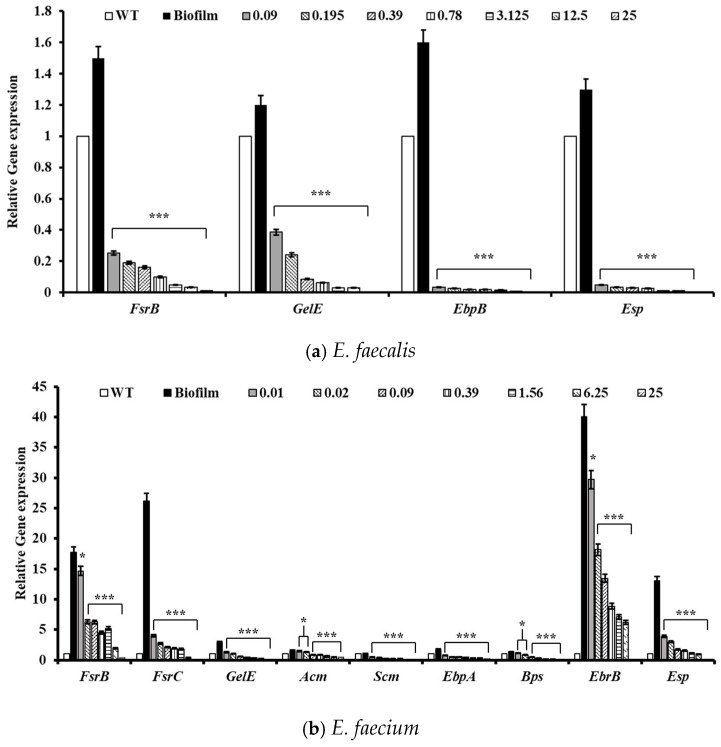
Gastrodin dose-dependently inhibited the expression of biofilm formation factors and quorum sensing coding genes in *E. faecalis* and *E. faecium*. (**a**) Gastrodin suppressed quorum sensing gene and biofilm formation gene (*fsrB*, *GelE*, *EbpB* and *Esp*) expression in *E. faecalis*. (**b**) Gastrodin suppressed quorum sensing gene and biofilm formation gene (*fsrB*, *fsrC*, *GelE*, *Acm*, *Scm*, *EbpA*, *Bps*, *EbrB*, and *Esp*) expression in *E. faecium*. Bacterial culture using gastrodin was performed using the same method as that used to confirm biofilm formation inhibition. WT is the planktonic growth cells. Biofilm is the negative control (* *p* < 0.05, *** *p* < 0.001).

Gastrodin dose-dependently repressed the expression of genes involved in biofilm formation (*Esp*, *EbpB*, *fsrB*, and *GelE*) of *E. faecalis* and (*fsrB*, *fsrC*, *GelE*, *Acm*, *Scm*, *EbpA*, *bps*, *EbrB*, and *Esp*) of *E. faecium* (Figure 5).

Therefore, these results suggest that gastrodin may inhibit biofilm formation by regulating the quorum sensing pathway.

### 2.5. Gastrodin Did Not Inhibit Bacterial Growth

Some antibacterial compounds also inhibit bacterial biofilm formation because antibiotics can kill the bacteria and indirectly reduce biofilm formation [27]. To determine whether the inhibition of biofilm formation by gastrodin occurs by inhibiting the growth of infectious bacteria or by directly inhibiting biofilm formation, bacterial growth was observed after treatment with 100 μg/mL of gastrodin.

**Figure 6:**
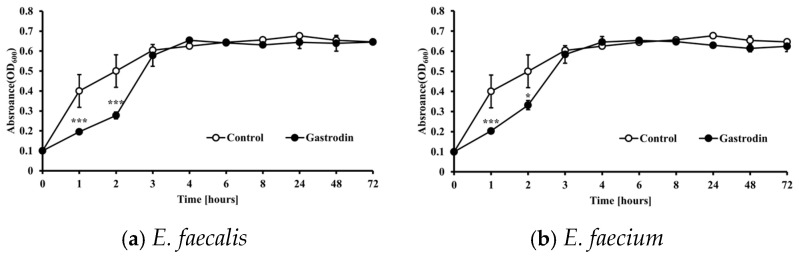
Gastrodin did not influence the growth of *E. faecalis* and *E. faecium*. (**a**) Gastrodin treatment did not affect the growth of *E. faecalis*. (**b**) Gastrodin treatment did not affect the growth of *E. faecium*. Gastrodin (100 μg/mL) was added to the OD_600_ = 1 bacterial cell and then incubated at 37 °C (* *p* < 0.05, *** *p* < 0.001).

Gastrodin did not inhibit the growth of *E. faecalis* and *E. faecium* (Figure 6).

## 3. Discussion

The present study addresses the urgent need for novel anti-infective strategies in the context of rising antimicrobial resistance. Rather than relying solely on bactericidal activity, we focused on the inhibition of biofilm formation, an important virulence trait that contributes to bacterial persistence, immune evasion, and reduced antibiotic susceptibility [8,9,16,28,29]. This approach is clinically relevant because biofilm-associated infections are often difficult to eradicate even when the causative organisms are not highly resistant in planktonic form.

In this study, gastrodin inhibited biofilm formation in multiple pathogenic species, with particularly strong activity against *E. faecium* and *C. acnes* and more moderate activity against *E. faecalis*, *S. aureus*, *S. sobrinus*, *P. aeruginosa*, and *E. coli*. The species-specific differences in sensitivity suggest that gastrodin may act on conserved yet context-dependent pathways involved in surface adhesion, extracellular matrix production, or quorum sensing regulation. Because biofilm development is governed by multiple overlapping regulatory networks, the distinct response patterns observed here may reflect differences in cell envelope composition, regulatory circuitry, and biofilm architecture among Gram-positive and Gram-negative bacteria.

A notable finding of this study is that gastrodin suppressed the expression of genes associated with the *fsr* quorum sensing system and biofilm-related virulence factors in *Enterococcus* spp. In *E. faecalis*, downregulation of *fsrB*, *gelE*, *cylLS*, *cylR2*, and *cylM* suggests that gastrodin may interfere with quorum sensing-dependent control of gelatinase production and cytolysin-associated virulence. In *E. faecium*, reduced expression of *fsrB*, *fsrC*, *gelE*, *esp*, *ebpA*, *ebpB*, *acm*, *scm*, *bps*, and *ebrB* indicates broader suppression of factors involved in adhesion, pili formation, and biofilm maturation. These results support the idea that gastrodin does not merely reduce biomass nonspecifically but instead modulates gene networks required for biofilm establishment and maintenance. This mechanism is consistent with previous studies reporting that plant-derived polyphenols and glycosides attenuate bacterial pathogenesis by disrupting quorum sensing networks [30,31].

The fact that gastrodin did not inhibit bacterial growth at the concentration tested is especially important. This suggests that the anti-biofilm effect is not simply a secondary consequence of bacteriostatic or bactericidal activity. Instead, gastrodin appears to function as an anti-virulence compound that selectively attenuates biofilm-associated phenotypes. Such an effect is advantageous for therapeutic development because it may impose less selective pressure for resistance than conventional antibiotics, while still weakening the pathogenic potential of the bacteria. This also makes gastrodin attractive as an adjuvant rather than a stand-alone antimicrobial agent.

The synergy observed between gastrodin and commercial antibiotics further strengthens this interpretation. Biofilm inhibition likely improves antibiotic access to bacterial cells and may also increase the metabolic state or vulnerability of the cells embedded within biofilms. In addition, suppression of quorum sensing- and adhesion-related genes may weaken the structural integrity of the biofilm matrix, making the bacterial community more susceptible to antibiotics. This is consistent with the general principle that anti-biofilm compounds can restore or potentiate antibiotic efficacy by disrupting protective multicellular structures [4,5,6,28,29]. In this context, gastrodin may be useful as a combination partner to improve the activity of existing antibiotics, particularly against enterococcal infections.

The low cytotoxicity observed in T24 and RAW 264.7 cells is also encouraging. A compound intended for anti-infective use must ideally show selective activity against pathogens without damaging host cells, and the present data suggest that gastrodin has a favorable preliminary safety profile. However, these results should be interpreted cautiously because in vitro cell viability assays cannot fully predict in vivo tolerability, pharmacokinetics, or tissue-specific toxicity [32]. Therefore, further evaluation in animal models will be necessary before considering translational applications.

This study has several limitations. First, the mechanistic analysis was limited to qRT-PCR, so the regulatory cascade affected by gastrodin was inferred at the transcript level only. Additional studies using proteomics, transcriptomics, mutant strains, or reporter assays would be valuable for defining the direct molecular target. Second, synergy was assessed by means of growth-based combination testing, but formal synergy metrics such as the fractional inhibitory concentration index should be included in future work. Third, the anti-biofilm effects were shown in vitro, and the clinical relevance of the findings should be confirmed in in vivo infection models, especially for device-associated or mucosal biofilm infections. Fourth, because the strongest effects were observed in *Enterococcus* spp., future studies should examine whether gastrodin is equally effective against resistant clinical isolates and mixed-species biofilms.

In summary, gastrodin appears to be a promising anti-virulence adjuvant that suppresses biofilm formation, downregulates quorum sensing- and adhesion-associated genes, and enhances the antibacterial activity of conventional antibiotics without evident host-cell toxicity. These findings provide a rationale for further preclinical development of gastrodin as a biofilm-targeting therapeutic candidate.

## 4. Materials and Methods

### 4.1. Materials and Bacteria Used in This Study

Gastrodin was purchased from Merck (Massachusetts, USA). The bacterial strains used in this study included *Enterococcus faecalis* (CCARM 5511), *Enterococcus faecium* (KACC11954), *Escherichia coli* (KACC11598), *Streptococcus mutans* (KACC16833), *Streptococcus sobrinus* (CCARM3506), *Staphylococcus aureus* (KCTC5809), *Pseudomonas aeruginosa* (KACC14021), *Cutibacterium acnes* (CCARM9009), and *Porphyromonas gingivalis* (KCTC5352) and are listed in Table 1. All bacterial strains were cultured at 37 °C. Aerobic bacteria (*E. faecalis*, *E. faecium*, *E. coli*, *S. mutans*, *S. sobrinus*, *S. aureus*, and *P. aeruginosa*) were cultured under aerobic conditions (37 °C, ambient atmosphere). Anaerobic bacteria (*C. acnes* and *P. gingivalis*) were maintained under anaerobic conditions (37 °C, anaerobic chamber: 5% CO_2_/10% H_2_/85% N_2_).

### 4.2. Biofilm Formation Assay

To determine whether gastrodin inhibited biofilm formation in the tested bacteria, biofilm formation was evaluated using a previously described crystal violet staining method with minor modifications [33].

Briefly, bacterial suspensions adjusted to OD_600_ = 0.1 were inoculated into 96-well flat-bottom plates containing the appropriate culture medium listed in Table 1. Gastrodin was serially diluted twofold to a maximum concentration of 100 μg/mL and added to the wells. The plates were incubated at 37 °C for 24 h. After incubation, the wells were washed twice with 200 μL PBS to remove planktonic cells, and 100 μL of 1% crystal violet solution was added to each well and incubated for 30 min. After staining, the wells were washed again with 200 μL PBS and destained with 150 μL of 30% acetic acid for 15 min. The absorbance of the released dye was measured at 595 nm using a microplate reader (Epoch, BioTek Instruments, Inc., Winooski, VT, USA). All experiments were performed in triplicate.

### 4.3. Synergic Antibacterial Effect

The synergistic antibacterial effects of gastrodin and antibiotics were assessed using a growth-based assay. Gastrodin was tested in combination with ampicillin, oxytetracycline, vancomycin, or streptomycin at the indicated concentrations. Bacterial inocula prepared as described in the biofilm assay were added to each treatment condition. After incubation at 37 °C for 24 h, bacterial growth was measured at 600 nm using a microplate reader (Epoch, BioTek Instruments, Inc., Winooski, VT, USA). The combination effect was evaluated by comparing the growth in combination-treated groups with that in groups treated with each compound alone [30].

### 4.4. MTT Assay for Human Cell Viability

T24 (human urinary bladder) and RAW 264.7 (murine macrophage) cell lines were used. Cells (10^4^ per well) were inoculated into 100 μL of RPMI 1640 and DMEM complete medium, respectively, in a 96-well plate and cultured at 37 °C for 24 h under 5% CO_2_ conditions to allow the cells to attach and recover before each treatment was performed. Thereafter, 100 μL of fresh complete medium containing the test compound, solvent control, or medium (untreated control) was added to each well. After incubating the plates for 24 h, the treatment medium was replaced with 100 μL of serum-free medium to minimize interference [34,35].

Next, 10 μL of 5 mg/mL MTT stock solution was added to each well and incubated for 2 h in a dark room at 37 °C under 5% CO_2_ conditions. After incubation, the supernatant was carefully removed, and 100 μL of solubilization solution (e.g., DMSO) was added to each well. The plate was shaken at room temperature using an orbital shaker for 10–15 min to completely dissolve the crystals, and then the absorbance was measured at 570 nm using a microplate reader (Epoch, BioTek Instruments, Inc., Winooski, VT, USA).

Viability was determined using the following equation:

Viability (%) = (Abs_sample/Abs_control) × 100

### 4.5. Gene Expression Analysis

Bacterial cultures treated with gastrodin were prepared using the same conditions as those used for the biofilm formation assay. Total RNA was isolated using TRIzol reagent (Thermo Fisher Scientific, Waltham, MA, USA) according to the manufacturer’s instructions, and cDNA was synthesized via reverse transcriptase (NanoHelix, Daejeon, Republic of Korea) reaction using 1 µg of RNA. qRT-PCR was performed using 2X SybrGreen qPCR Master Mix (CellSafe, Yongin, Republic of Korea). Primer sequences are listed in Table 2. The 23sRNA and the tufA gene were used as internal controls. qRT-PCR data were analyzed using the relative quantification (2^−ΔΔCt^) method [33,36,37,38,39].

### 4.6. Bacterial Growth Assay

Bacterial growth curves were determined as previously described with slight modifications [40]. *E. faecalis* and *E. faecium* cultures were prepared with tryptic soy broth at an OD_600_ = 0.1. Gastrodin (100 μg/mL) was added to each culture, and the cells were incubated at 37 °C. Bacterial growth was monitored by measuring the absorbance at 600 nm using a microplate reader at the indicated time [40].

### 4.7. Statistical Analysis

All experiments were performed at least three times independently, and the data are presented as the mean ± standard deviation. Statistical significance was determined using one-way ANOVA followed by Tukey’s post hoc test. A value of *p* < 0.05 was considered statistically significant. Statistical annotations are denoted as * *p* < 0.05, ** *p* < 0.01, and *** *p* < 0.001.

## 5. Conclusions

These results show that gastrodin has good potential as a novel antimicrobial adjuvant candidate for biofilm-associated bacterial infections; however, further validation, including in vivo studies, is required to confirm its efficacy and therapeutic potential. In this study, gastrodin effectively inhibited biofilm formation in multiple pathogenic species, including *E. faecalis*, *E. faecium*, and *S. aureus*, suppressed the expression of quorum sensing genes (*fsrB*, *fsrC*, *gelE*), and demonstrated synergistic antibacterial activity in combination with conventional antibiotics without cytotoxicity to human cells. The proposed mechanism involves downregulation of the *fsr* quorum sensing cascade, which controls biofilm-associated virulence gene expression. These findings support gastrodin as a promising anti-virulence adjuvant for biofilm-associated infections. However, the molecular mechanism was assessed solely by means of qPCR; more comprehensive analyses such as transcriptomic or proteomic studies are needed for further validation. Future studies should include in vivo infection models, FICI-based synergy confirmation, and broader investigation against resistant clinical strains.

## Figures and Tables

**Table 1:** Bacteria used in this study.

Strain	Strain Number	Medium Used in This Study	Source
*Enterococcus faecalis*	CCARM5511	TSB	Purchased from KACC (Korean Agricultural Culture Collection), CCARM (Culture Collection of Antimicrobial Resistant Microbes), or KCTC (Korean Collection for Type Cultures)
*Enterococcus faecium*	KACC11954	TSB	
*Escherichia coli*	KACC11598	LB	
*Streptococcus mutans*	KACC16833	TSB	
*Streptococcus sobrinus*	CCARM3506	BHI	
*Staphylococcus aureus*	KCTC5809	TSB	
*Pseudomonas aeruginosa*	KACC14021	LB	
*Cutibacterium acnes*	CCARM9009	BHI	
*Porphyromonas gingivalis*	KCTC5352	TSB	

TSB: Tryptic soy broth, LB: Luria–Bertani Medium, BHI: Brain heart infusion.

**Table 2:** Primers used for real-time RT-PCR.

Genes	Primer Sequence: 5′ to 3′	Function	Reference
For *E. faecium*			
*esp*	F: CCACGAGTTAGAGGGAACAG R: TTGGAGCCCCATCTTTTTCA	Biofilm formation	[39]
*bps*	F: TATCAGCAACAAGCGGTCAA R: AATCCTGCCCTTTTTCGATT	Biofilm formation	[37]
*fsrC*	F: GCTTATTTGGAAGAACAACGTATCAA R: CGAAACATCGCTAGCTCTTCGT	Efae regulator	[38]
*gelE*	F: CGGAACATACTGCCGGTTTAGA R: TGGATTAGATGCCACCCGAAAT	Gelatinase	[38]
*fsrB*	F: TGCTCAAAAAGCAAAGCCTTATAA R: GATGACGAGACCGTAGAGTATTACTGAA	Efae regulator	[38]
*ebpA*	F: ACCAAGCCAGACGAAATAGAAGAAG R: ATTGTTTTGGTCAGGTGCATCATAGA	Biofilm-associated pili	[37]
*acm*	F: TCAGCAGTAATGTCACTTCGTTG R: GAATAGGCTGTTCATCTGCTCG	Gelatinase	[36]
*scm*	F: CTAACTGGTAACTATGGCTTGT R: GTCCGTGCTGTCACTTGT	Gelatinase	[36]
*tufA*	F: TACACGCCACTACGCTCAC R: AGCTCCGTCCATTTGAGCAG	Housekeeping gene	[39]
For *E. faecalis*			
*gelE*	F: CGFAACATACTCAACGTTTGAC R: TGGATTAGATGCADDDGAAAT	Gelatinase	[33]
*esp*	F: GCATCAGTATTAGTTGGT R: TTCCTTGTAACACATCAC	Biofilm formation	[33]
*fsrB*	F: TGCYCAAAAAGCAAAGCCTTATAA R: GATGACGAGACCGTAGAGTATTACTGAA	Efae regulator	[33]
*ebpB*	F: CGTACAGGAGGCAAGTCTTT R: AGGTATTCCCCGCTTGATTT	Biofilm-associated pili	[33]
*cylLS*	F: CTGTTGCGGCGACAGCT R: CCACCAACCCAGCCACAA	Cytolysin toxin	[33]
*cylR2*	F: TTTATTTTTATTGGATATCATTCTGTAGTC R: TTCGCTCATCTTTTTTGAATACAG	Cytolysin regulatory	[33]
*cylM*	F: TCGGACACGGTATATATAGCTATGT R: TTCTACTAGTGTACTTTGATTACCATAATAATT	Cytolysin toxin	[33]
23s RNA gene	F: CCTATCGGCCTCGGCTTAG R: AGCGAAAGACAGGTGAGAATCC	Housekeeping gene	[33]

## Data Availability

Data will be provided upon request.

## Abbreviations

The following abbreviations are used in this manuscript:

qRT-PCR	Quantitative Reverse Transcription Polymerase Chain Reaction
IC_50_	Half Maximal Inhibitory Concentration
Amp	Ampicillin
Oxy	Oxytetracycline
Van	Vancomycin
Stre	Streptomycin
Gas	Gastrodin
QS system	Quorum Sensing system
KACC	Korean Agricultural Culture Collection
CCARM	Culture Collection of Antimicrobial Resistant Microbes
KCTC	Korean Collection for Type Cultures
TSB	Tryptic soy broth
LB	Luria–Bertani Medium
BHI	Brain heart infusion
